# USING THE SAMHSA TRAUMA-INFORMED PRINCIPLES TO ADAPT ADVANCE CARE PLANNING FOR OLDER ADULTS WITH LOW SES

**DOI:** 10.1093/geroni/igae098.4390

**Published:** 2024-12-31

**Authors:** Christine Kimpel, Lorely Chavez, Christian Ketel, Kate Clouse

**Affiliations:** Vanderbilt University, Nashville, Tennessee, United States; Vanderbilt University, Nashville, Tennessee, United States; Vanderbilt University, Nashville, Tennessee, United States; Vanderbilt University, Nashville, Tennessee, United States

## Abstract

Advance care planning (ACP) is the process of preparing for future medical care received during times of cognitive incapacity, but the process may be very stressful for low SES communities. To address lifetime traumas and the everyday stress of unmet needs, we used the Substance Abuse and Mental Health Services Administration’s (SAMHSA) trauma-informed principles to adapt an ACP intervention. Using a single-arm pre-post intervention design and mixed methods approach, we continually adapted the intervention with participant feedback (n=19). The initial intervention consisted of an audio-recorded, one-time, in-person, flexible conversational approach using a discussion prompt checklist, conducted in a quiet private setting in a subsidized housing community. We thematically analyzed field notes and intervention transcripts using the SAMHSA framework. Nineteen participants completed the intervention (median age: 60, IQR: 58-63): 10 identified as Black or African American and 12 as men. Themes included trauma awareness (safety), facilitating privacy (trustworthiness), peer referral (peer support), person-centered communication (collaboration), healthcare risk-benefit analysis education (empowerment), and acknowledgment of historical trauma, stigma, and access challenges (equity). We have found the SAMHSA framework useful for enhancing trauma awareness during the ACP process. There is significant conceptual overlap between the person-centeredness of ACP and the SAMHSA principles. The universal safety precautions help to avoid re-traumatization while discussing previous death-related experiences. The equity focus provides opportunities to tailor the approach. These trauma-informed principles should be implemented in clinical provider training, organizational policy and procedures, and future research interventions with similar communities.

